# Choroidal Melanoma Metastatic to the Contralateral Medial Rectus After Orbital Exenteration

**DOI:** 10.4274/tjo.galenos.2019.35589

**Published:** 2019-10-24

**Authors:** Elizabeth McElnea, Louis J. Stevenson, Cesar Salinas La Rosa, Sem Liew, Thomas G. Hardy

**Affiliations:** 1 Royal Victorian Eye and Ear Hospital, Orbit, Plastics and Lacrimal Unit, East Melbourne, Victoria, Australia; 2 St. Vincent’s University Hospital, Anatomical Pathology Department, Fitzroy, Victoria, Australia; 3 Victorian Oncology Care, Berwick, Victoria, Australia

**Keywords:** Melanoma, orbital metastasis, exenteration, cancer

## Abstract

A 78-year-old Caucasian woman presented with pain in her right and only eye that was worse on abduction. Her history was significant for a choroidal melanoma affecting her left eye for which she underwent an orbital exenteration 12 years previously. Computed tomography and magnetic resonance imaging of the right orbit identified a mass lesion affecting the medial rectus, suspicious for metastatic melanoma. A histopathological diagnosis of metastatic melanoma was subsequently made following biopsy of the right medial rectus.

## Introduction

Metastases to the orbit are rare, comprising 1-13% of all orbital tumors and occurring in 2-3% of cancer patients.^1^ Most are carcinomas and over 90% are unilateral.^1^

Melanoma represents 5.3-15% of all metastases to the orbit.^1,2,3,4^ Primary sites include the skin and uveal tract, but may be unidentifiable in some cases.^3^ In one review, the primary tumor was a cutaneous melanoma in 5 cases, a uveal melanoma in the contralateral eye in 1 case, and was unidentified in another case.^2^

Melanoma may tend to metastasize to the extraocular muscles.^3,5,6^ There are two reports of bilateral extraocular muscle metastases from uveal melanoma^7,8^ and three reports of bilateral extraocular muscle metastases from non-uveal melanoma.^9,10,11^ Tumor was found in one or several extraocular muscles in 4 of 7 cases (57%)^2^ and in 8 of 29 cases (28%)^5^ in two reviews of melanoma metastases to the orbit.

We describe an unusual case of choroidal melanoma metastatic to the contralateral medial rectus 12 years after orbital exenteration for extrascleral choroidal melanoma as an illustration of the important clinical features of metastatic malignant melanoma to the extraocular muscles.

## Case Report

A 78-year-old Caucasian woman presented with a 3-week history of pain behind her right and only eye that worsened when looking to her right. She had not noticed a change in her appearance.

Twelve years previously she had undergone orbital exenteration with postoperative radiotherapy for extrascleral spread of left choroidal melanoma. Five years after this, surveillance imaging identified an FDG-avid mass in her right kidney. Radical nephrectomy confirmed metastatic choroidal melanoma. Postoperative imaging did not show residual or distant metastatic disease. She visited her oncologist regularly for follow-up and had a positron emission tomography (PET) scan every 4 months.

Her past ocular history was also significant for a right hemiretinal vein occlusion with cystoid macular edema, for which she received treatment with ranibizumab.

Her past medical history was significant for hypertension, hypercholesterolemia, and gastroesophageal reflux disease. Her medications were candesartan, atorvastatin, and rabeprozole.

**Figure 1:**
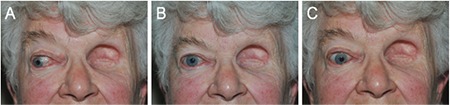
Clinical photographs showing right eye in abduction (A), primary gaze (B), and adduction (C)

On examination her Snellen visual acuity was 6/5. Her anterior segment was normal. Fundoscopy showed signs of prior hemiretinal vein occlusion without cystoid macular edema. Intraocular pressure was 15 mmHg. Optic nerve function was intact. Adduction was limited (Figure 1) and abduction was painful.

**Figure 2:**
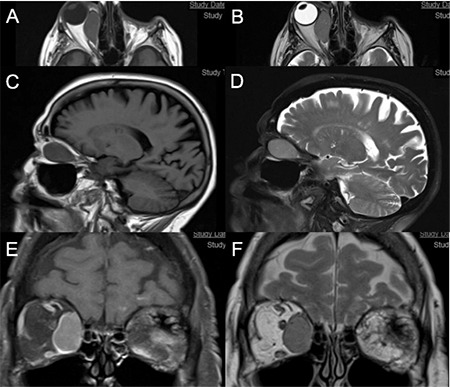
Magnetic resonance imaging of our patient demonstrating a large mass in the right medial rectus: Axial T1 (A) and T2 (B); sagittal T1 (C) and T2 (D); coronal T1, fat suppressed (E), and T2 (F). In general, the mass is hyperintense in T1-weighted images and hypointense in T2-weighted images

Orbital computed tomography (CT) showed homogenous, fusiform enlargement of the right medial rectus muscle which involved its tendon. Magnetic resonance imaging (MRI) showed hyperintensity of the medial rectus on T1-weighted imaging and hypointensity on T2-weighted imaging (Figure 2) compatible with melanoma metastases. PET did not show any further metastases elsewhere.

**Figure 3:**
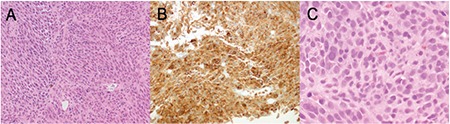
Histopathological examination of muscle biopsy. (A) Hematoxylin and eosin stain, 200X; diffusely infiltrative nested malignant cells, some with intracytoplasmic brown pigment; (B) Melan A immunohistochemistry stain, 200X; diffusely positive staining consistent with melanoma; (C) hematoxylin and eosin stain, 600X; abnormal plump spindle and epithelioid cells showing nuclear pleomorphism with some nuclei bearing inclusions and others intracytoplasmic pigment consistent with melanoma cells (C).

Under general anesthetic, she underwent right medial rectus biopsy. An incision was made behind the caruncle and dissection to the medial rectus was performed. After the biopsy sample was retrieved and hemostasis achieved, the incision at the caruncle was closed with two 6/0 vicryl interrupted sutures. A dark brown vascular lesion within the muscle was noted. Histopathological examination revealed this to be metastatic malignant melanoma (Figure 3). Next-generation sequencing identified a somatic mutation in the *GNA11* gene. A sensitizing BRAF mutation was not found in this tumor.

The patient commenced dual immunotherapy with ipilimumab and nivolumab. Following three cycles of such treatment, she developed immune-related enteritis and pneumonitis necessitating intensive care unit admission, ventilation, and treatment with high-dose intravenous corticosteroids. Though she recovered well medically, MRI demonstrated disease progression in the right orbit, to which she subsequently received 36 Gy stereotactic radiotherapy. Repeat MRI and PET showed no regression of the lesion. She is currently being re-challenged with nivolumab. It is now 16 months following the diagnosis of right orbital disease.

## Discussion

The commonest malignancies to metastasize to the eye and orbit are breast, lung, unknown primary, and prostate cancers, which together account for 75% of all such metastases.^1,3,4,12,13^ Cutaneous malignant melanoma is the ninth most common cancer^14^, the fifth most common source of ophthalmic metastases, and may account for up to 15% of metastases to the orbit.^2^

Proptosis (58%) and diplopia (54%) are the most common presenting symptoms of orbital metastases.^5^ Pain, as in the case described here, is also recognized as a presenting symptom and may be related to scleral indentation by the tumor. Some patients present with eyelid abnormalities such as ptosis or distorted lid margin. Chemosis and conjunctival injection are presenting features in others and may indicate vascular congestion or an inflammatory response. Fewer patients present with visual disturbances such as reduced vision and/or metamorphopsia, which may be related to choroidal folds and/or papilledema.^5^

In one study, 24 patients with primary cutaneous melanoma developed orbital metastases at a mean of 3.3 years after initial diagnosis.^5^ In another review, the mean interval from the diagnosis of the primary tumor to the diagnosis of orbital metastases was 7.8 years for choroidal melanoma and 5.5 years for cutaneous melanoma.^2^ The interval from the diagnosis of our patient’s choroidal melanoma to the onset of her ocular symptoms was 12 years, which is somewhat longer than the mean reported in each of these studies. Many studies have reported shorter intervals between diagnosis of the primary tumor and the development of intraocular metastases relative to the development of orbital metastases.^6,15^

On MRI, the extraocular muscle in our patient showed T1 signal hyperintensity and T2 signal hypointensity, which is consistent with the paramagnetic properties of melanin found in malignant melanoma.^7^ Due to the incidence of concurrent metastases, patients suspected of having metastatic melanoma should be evaluated by total body PET, as with our patient.^16^

A higher predilection for muscle of metastatic melanoma compared with carcinoma has been reported in several reviews.^3,5,6^ This muscle tropism of orbital metastatic melanoma explains why diplopia is one of the main presenting symptoms and limited ocular motility the main presenting sign of extraocular muscle involvement with metastases. In one reported series, the metastasis was situated in the extraocular muscle in 4 of 7 cases of metastatic melanoma to the orbit.^2^ Tumor adhesion molecules may play a role in such site-specific metastases to the orbit.^17^

The differential diagnosis of extraocular muscle enlargement includes thyroid-associated orbitopathy, lymphoproliferative disease (especially lymphoma), inflammatory orbital disease (orbital myositis, IgG4-related disease, idiopathic orbital inflammation), acromegaly, vascular and infectious causes, and metastatic malignancy.^9,18,19^ Accurate diagnosis can be difficult; some reports describe the clinical picture of extraocular muscle metastases as being very similar to that of thyroid eye disease with imaging demonstrating selective enlargement of the medial and lateral rectus muscles.^7,10,11,20^ In the case described here, diagnosis was based on clinical findings of weakness of the affected muscle and atypical extraocular muscle enlargement in the context of previously metastatic choroidal melanoma.

Choroidal melanoma may infiltrate the extraocular muscles following metastasis, as in the case described here. In a review of 1842 cases of choroidal melanoma, there was recurrence of tumor in the orbit following simple enucleation in 55 cases (3%).^21^ Forty-three of these occurred in the group of 235 patients in which the original histopathological sections revealed evidence of extrascleral extension, while only 12 of the 1607 patients without evidence of the same developed orbital recurrence. Put another way, the chance of a patient having orbital recurrence was 26 times greater if extrascleral extension of the initial tumor was noted.^21^ The orbital recurrence rate was 65% for those cases in which the extraocular tumor had a cross-sectional area of 100 mm^2^, and one-fifth of that rate when the extrascleral extension of the tumor was smaller than this.^21^ In cases showing no evidence of encapsulation or in which there was evidence that the surgeon had cut into the epibulbar tumor, the recurrence rate was 6 times greater than when there was no evidence of the same.^21^

In the 12 cases with recurrence in the orbit without demonstrable extrascleral extension at the time of enucleation, it is worth noting that 2 patients had had surgery for retinal detachment, 1 for glaucoma, and another had experienced traumatic rupture of the globe. It is thought that approximately 2 of every 5 cases of choroidal melanoma have extraocular extension when enucleation is preceded by retinal detachment surgery.^22^ Tumor cells may be released into the orbit during drainage of subretinal fluid or there may be gross extension of tumor through the scleral wound to the orbit. Inadvertent seeding during other intraocular surgeries or following globe rupture might also be expected to occur.

At least 2 cases of choroidal melanoma treated with evisceration because of an erroneous preoperative diagnosis of panophthalmitis have been described.^21^ Both patients experienced recurrence of tumor within the scleral shell and subsequent extrascleral extension to the orbit. One patient underwent exenteration 4.3 years after evisceration and survived 4 months while the second underwent exenteration 5 years after evisceration and died 2 years later.^21^ Ten percent of blind, painful eyes with opaque media were found to contain unsuspected malignant neoplasms, usually uveal melanomas, on pathological examination.^23,24^ Consequently, the presence of a malignant intraocular neoplasm should be excluded prior to evisceration of any eye, particularly those with opaque media, and if this cannot be done confidently, enucleation should be performed.^25^

Orbital metastases are rarely the first sign of metastatic melanoma but generally occur in patients already having multiple metastases.^2^ It is much less common for ocular metastases to be the first evidence of disease spread.^26^ In one study, 68 of 76 patients (89%) with primary cutaneous melanoma had at least one other non-ophthalmic distant metastasis at the time of presentation with ophthalmic metastasis.^5^ These were cutaneous and/or subcutaneous in 45%, lymph nodes in 38%, central nervous system in 34%, lungs in 27%, and liver in 25%. The remaining 8 patients with negative metastatic evaluations were all later diagnosed with non-ophthalmic systemic metastases.^5^

The median survival times of those with hepatic metastases from choroidal melanoma is typically less than a year, but patients with only extrahepatic metastases appear to have longer median survival times of 19-28 months.^27,28^ In one study, patients with orbital metastases from cutaneous melanoma survived an average of 7.5 months, whereas those with intraocular metastases survived 6.6 months.^5^ Zografos et al.^2^ reviewed 14 cases of melanoma metastatic from various sites (cutaneous in 5 cases, uveal in 3 cases, mucosal in 1 case, and unknown primary in 2 cases) and found that patients with orbital metastases still had better survival times than those with intraocular metastases, at 19.7 (range 5-48 months) and 8.8. months, respectively. Ninety percent of patients with any ophthalmic metastasis from melanoma do not survive beyond 12 months.^2^

Symptom palliation is, consequently, often the main goal of the management of orbital metastatic melanoma and aims to maximize ocular function while minimizing discomfort. The correction of bothersome diplopia, reduction of proptosis that may be unsightly and/or prevent eyelid closure and thus lead to exposure keratopathy, or treatment of optic nerve compression to maintain or restore visual function must often be considered.^2^ The choice of treatment modality (surgery, chemotherapy, or radiotherapy) will depend on the symptomatology, treatment toxicities, and the patient’s general health and life expectancy.

Solitary orbital metastases can be treated with surgery and radiation with or without chemotherapy or immunotherapy. The goal of surgery is to decrease tumor volume, though significant debulking of extraocular muscle metastases is often not possible. In our case, complete local control would not have been possible given the extent of muscle involvement. Radiation may be the primary mode of treatment in the absence of other effective options or may be used to address residual microscopic disease, but is generally not the treatment modality of choice for choroidal melanoma. In a review of patients with exclusively extraocular muscle metastases, the most common treatment was excision of the tumor mass, which was conducted in 11 of 19 patients (58%). Radiation was used in 9 (47%) of these patients. The treatment advances that have improved survival in patients with cutaneous melanoma have unfortunately not provided similar benefits in those with advanced choroidal melanoma.

This unique case highlights the possibility of recurrence of uveal melanoma metastases in unusual locations in survivors, particularly survivors of metastatic disease elsewhere.^8^ Proptosis, diplopia, pain, and eyelid changes are the most common presenting symptoms of orbital metastases. At the time of their presentation with ophthalmic metastatic disease, most patients either have previously diagnosed widespread systemic disease or disseminated disease is discovered upon work up for metastatic disease. Patient survival largely depends on the extent of systemic disease and is generally not very long, rarely over one year.^2^
